# Promoter hypermethylation of *KLOTHO*; an anti-senescence related gene in colorectal cancer patients of Kashmir valley

**Published:** 2015-12

**Authors:** Malik Perveez, Malik Ajaz, Dil Afroze

**Affiliations:** 1 Department of General and Minimal Access Surgery, SKIMS Srinagar, India; 2 Department of Immunology and Molecular Medicine, SKIMS Srinagar, India

**Keywords:** *KLOTHO*, Promoter hypermethylation, Colorectal cancer, Tumor suppressor

## Abstract

Hypermethylation of CpG islands located in the promoter regions of genes is a major event in the development of the majority of cancer types, due to the subsequent aberrant silencing of important tumor suppressor genes. *KLOTHO*; a novel gene associated primarily with suppressing senescence has been shown to contribute to tumorigenesis as a result of its impaired function. Recently the relevance of *KLOTHO *promoter hypermethylation in colorectal carcinoma in humans has been reported. We analyzed the promoter hypermethylation of *KLOTHO *gene in 50 histopathologically confirmed tumor and adjacent normal tissues of colorectal cancer patients. Methylation was assessed by bisulfite conversion of DNA followed by methylation specific-polymerase chain reaction. Methylation status was compared with gender, smoking status and histopathological parameters of patients. Promoter hypermethylation in *KLOTHO *gene was detected in 86% (43/50) of tumor tissues and 14% (7/50) of adjacent normal tissues. The methylation pattern differed significantly between tumor and adjacent normal tissues (*P*<0.0001). However, no association was found between promoter hypermethylation status and gender (*P*=0.68), smoking status (*P*=0.64) or other histopathological parameters (*P>*0.05) of colorectal cancer patients. We conclude that this novel tumor suppressor gene is epigenetically inactivated in colorectal cancer in our population paving way towards the potential of *KLOTHO *promoter hypermethylation as a predictor of the prognosis in colorectal cancer patients.

## INTRODUCTION

Globally, 12 percent of deaths are attributed to cancer annually, with lung and breast cancers being the most common in men and women, respectively [1]. Most frequent cancers account for 2 million deaths and 3 million new cases each year comprise of the cancers of the gastrointestinal tract [1]. Colorectal cancer (CRC) is the third most commonly diagnosed cancer in men and the second most common in women [2, 3]. The prevalence of CRC varies considerably across geographical locations. Kashmir valley, the northernmost area of India has been reported as a high incidence region of gastrointestinal cancers [4, 5]. In Kashmir valley, CRC is the third commongastrointestinal tract cancer [6]. The human *KLOTHO *gene is a 5-exon gene located on chromosome 13q12 spanning a region of around 50 kb [7]. Recently, *KLOTHO *was identified as a potential tumor suppressor and a candidate target for epigenetic silencing in various cancers [8-11]. Abnormal activation of the canonical Wnt pathway due to epigenetic deregulation of Wnt antagonists is thought to play a critical role in tumorigenesis [12]. *KLOTHO *was reported to function as a secreted Wnt antagonist and as a tumor suppressor. Epigenetic alterations leading to gene silencing of secreted Wnt antagonists is considered a common incident in various human malignancies. *KLOTHO *has been shown to inhibit Wnt signaling activity by forming a complex with Wnt3, which is similar to that of the secreted Frizzled-related proteins (SFRP) class of Wnt antagonists [13, 14]. Silencing *KLOTHO *gene expression is mainly mediated through promoter hypermethylation and histone deacetylation in cancer. Recent studies have elucidated the relevance of *KLOTHO *promoter hypermethylation in colorectal carcinoma in humans [9, 15].This study was aimed to analyze the promoter hypermethylation of *KLOTHO *gene and correlate this epigenetic change with various clinico-histopathological parameters in subjects with colorectal carcinoma.

## MATERIALS AND METHODS

The study was conducted in the Department of General & Minimal invasive Surgery and Department of Immunology & Molecular Medicine, Sher-i-Kashmir Institute of Medical Sciences (SKIMS), India. The research work was initiated following the approval by the Institutional Ethics Committee. Protocol followed for the study was in conformity with the Declaration of Helsinki developed by World Medical Association. The study comprised of total 50 patients with colorectal carcinoma belonging to the ethnic population of Kashmir valley. Patients consenting to participate willingly were included in the study. A well drafted questionnaire was used to collect information from the study subjects. Histopathologically confirmed tumor specimens from resected tissues and adjacent normal tissues were analyzed for hypermethylation status in the promoter region of *KLOTHO *gene. Tissue specimens were collected in sterile polypropylene vials and stored at -70ºC. The vials were properly labeled to prevent possible mixing of the specimens and for easy retrieval of the required sample.

Promoter methylation status of *KLOTHO *gene was assessed by methylation specific- polymerase chain reaction (MS-PCR). Genomic DNA was bisulfite treated using EZ- DNA Methylation Direct™ Kit (Zymo Research) as per protocol provided by the manufacturer.

**Figure 1:**
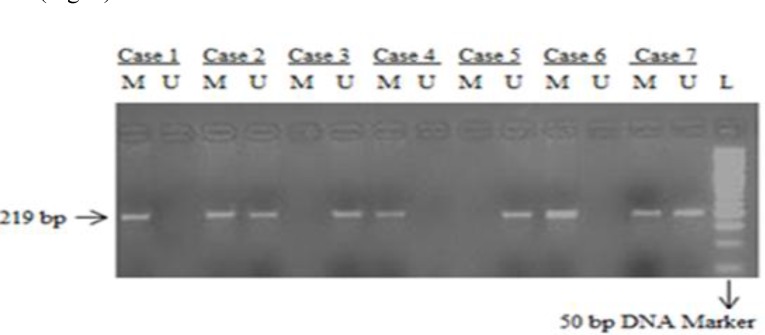
Agarose gel electrophoresis picture representing MS-PCR of *KLOTHO* in colorectal carcinoma

Methylation in 219 bp promoter region of *KLOTHO *gene was assessed by using the bisulfite treated DNA as template for MS-PCR using methylation specific primers as described earlier [16]. Two primer pairs discriminating between the methylated and non- methylated DNA were used. For each sample two PCR reactions were set with each primer pair. Successful amplification with either of the two pairs indicated the methylation or non-methylation in the promoter region. An aliquot of each PCR product was separated on 2% agarose gel. The gel was stained with ethidium bromide andphotographed under UV illumination. The reproducibility of the results was confirmed by repeating MSP analysis for each DNA sample and using unmodified DNA template as control (Fig. 1).

Statistical analysis was performed using SPSS software (Version 20). Fisher’s exact test, Chi Square test for homogeneity of proportions and Odds ratio was used wherever applicable. Statistical significance was considered at *P*< 0.05.

## RESULTS

Mean age of cases was 51.2±14.3 years in colorectal carcinoma patients, 30% (15) of patients were in the age group > 60 years representing the majority of cases in our study. Colorectal carcinoma was least common below 30 years of age. Among the studied subjects; 56% (28) were males and 44% (35) were females with male: female ratio of 1.27.

In our study group most common clinical symptom at presentation was bleeding per rectum (n=35), followed by change in bowel habits (n=28), easy fatigability (n=16), pain abdomen (n=12), generalized weakness and tenesmus in 7 cases. We observed a positive family history of 24% (12/50) in our study whereas positive smoking status corresponded to 72% (36/50). The most common site of lesion was rectum (n=14), followed by sigmoid colon (n=13), ascending colon (n=8), descending colon (n=5), transverse (n=4). Both splenic and hepatic flexure comprised of 3 cases each. Histopathological examination revealed that the majority of cases (27/50) had well differentiated, followed by poorly differentiated (10/50) and moderately differentiated (8/50) tumor histopathology. Signet ring type and mucinous types corresponded to 4/50 and 1/50 respectively.

**Table 1:** Relationship between methylation status and age in colorectal carcinoma

**Age Groups (Years)**	Methylated		Unmethylated		P
	N %		N %		
21-30	6	13.95	1	14.28	
31-40	7	16.27	2	28.57	0.59
41-50	6	13.95	1	14.28	1.0
51-60	11	25.58	1	14.28	0.70
>60	13	30.23	2	28.57	0.93

**Table 2:** Relationship of smoking and methylation status in colorectal carcinoma

**Smoking**	**Methylation Present**		**Methylation Absent**		**OR (95%CI)**	**P**
	**N**	**%**	**N**	**%**		
**No**	12	85.72	2	14.28	1.0	-
**Yes**	31	86.12	5	13.88	1.03 (0.17-0.60)	0.64

**Table 3:** Relationship between methylation status and histopathology in colorectal carcinoma

**Histopathology**	**Methylation status**		**OR (95%CT)** **(95% CI)**	***P***
	**Present**	**Absent**		
**Well differentiated Adenocarcinoma**	24	3	1.0	1
**Moderately differentiated Adenocarcinoma**	7	1	1.14(0.10-12.7)	0.66
**Poorly differentiated Adenocarcinoma**	8	2	2(0.28-14.1)	0.41
**Mucinous type**	1	0	NA	NA
**Signet ring type**	3	1	2.66(0.20-34.5)	0.90

We assessed promoter hypermethylation of *KLOTHO *in 50 colorectal carcinoma tissues and in adjacent normal tissues as controls. Notably, *KLOTHO *promoter hypermethylation was detected in 86% (43/50) tumor tissues and 14% (7/50) adjacent normal tissues. The methylation status was significantly different in tumor versus normal tissue (*P*<0.0001). The relationship between various clinico-histopathological parameters and promoter hypermethylation of *KLOTHO *gene are shown in Tables 1-3.

## DISCUSSION

DNA methylation plays a fundamental role in cell cycle regulation, embryonic development, gene imprinting and gene silencing [17]. Aberrant methylation of normally unmethylated CpG islands has been connected to transcriptional inactivation [18]. Abnormal hypermethylation in promoter CpG islands of genes, resulting in transcriptional silencing, has been widely associated with cancers [19]. Promoter hypermethylation can originate very early in tumor progression and is one of the hallmarks of carcinogenesis concomitant to transcriptional silencing and loss of expression in genes encoding several cellular pathways [18]. Senescence-related DNA damage is linked to many cancers [20]. The relationship between senescence and tumorigenesis is close but intricate [20, 21].

*KLOTHO *has been reported to be a novel gene suppressing senescence [22, 23]. Recently, growing evidences demonstrated that impaired *KLOTHO *function may contribute to tumorigenesis. Various studies have observed that promoter DNA hypermethylation contributes to *KLOTHO *silencing and down-regulation in many cancers [8, 10, 11, 14]. In view of these observations, we conducted this prospective study to analyze the promoter hypermethylation status of *KLOTHO *gene in colorectal cancer patients and elucidate its association with various clinic-pathological parameters. In our study population, 56% of cases with colorectal carcinoma were males and 44% were females with male: female ratio of 1.27. This was consistent with the study conducted by Javed *et al, *who reported a male-female ratio of 1.2 in colorectal carcinoma [6]. Despite an increasing cancer incidence in both genders, the effects of sex on colorectal cancer are well established and women are less likely to develop cancers than men [24]. The incidence of colorectal cancer throughout different populations shows male dominance with a constant male-to-female ratio. This sex ratio cannot be completely attributed to the disparities in the prevalence of known risk factors between the sexes [25]. Recent advances in the molecular biology of colorectal cancer have led to greater understanding of the effect of estrogen in carcinogenesis. Estrogen has a potentially protective effect against the development of colorectal cancer [26]. In our study 30% of patients had more than 60years of age, representing the majority of cases. Colorectal carcinoma was least common below 30 years of age. Incidence of cancers usually correlates with advancing age. An epidemiological study conducted to determine the incidence of CRC in Kashmir reported highest frequency in the age groups 55-59 followed by 65-69 years [6]. We observed a positive family history of 24% in our study. Of common malignancies, CRC has one of the largest proportions of familial cases. Kindred and twin studies estimated that approximately 30% of all CRC cases are an inherited form of the disease [27]. Approximately 5% of cases are associated with highly penetrant inherited mutations and clinical presentations that have been well characterized. The etiologies of the remaining 20–30% of inherited CRCs are not completely understood. Studies on first-degree relatives of patients have showed conflicting outcomes concerning the risk of colorectal cancer [28, 29]. We also observed a high frequency (72%) of positive smoking status in colorectal carcinoma patients (36/50). Botteri *et al*, observed an increased risk associated with smoking in both men and women, the positive association with smoking was more evident for rectal cancer compared to colon cancer [30].

Methylation in promoter region of *KLOTHO *was analyzed by MS-PCR in colorectal carcinoma patients. Promoter hypermethylation was detected in 86% tumor tissues and 14% adjacent normal tissues. There was significantly higher methylation in tumor compared to normal tissue (*P*<0.0001). A study analyzing the role of *KLOTHO *promoter methylation and colorectal carcinoma conducted by Pan *et al, *reported a methylation frequency of 85% and partial or no methylation in adjacent normal tissues [9]. They also reported frequent methylation in colon cancer cells by MSP analysis. A similar study by Gan *et al*, reported a methylation frequency of 76% in colorectal carcinoma tissues [15]. The comparison between clino-histopthological parameters of colorectal carcinoma patients with methylation of *KLOTHO *promoter showed no significant association which was in conformity by the study conducted by Pan *et al*, [9]. The study elucidates an intricate role of *KLOTHO *promoter hypermethylation in colorectal carcinoma irrespective of any discernible role in clinical and histo-pathological parameters. Thus *KLOTHO *promoter hypermethylation can be used as an independent prognosis factor to predict the outcome of colorectal carcinoma patients, however larger study patients and follow up study is needed to substantiate the results of this study.

## Notes

**Acknowledgements: **The authors are thankful to the *Department of General &Minimal Access Surgery *and *Department of Immunology & Molecular Medicine *where the experimental work was carried. The authors also acknowledge the financial support in the form of research grant provided by Sher-i-Kashmir institute of Medical Sciences (SKIMS), Srinagar for undertaking this study.
